# High-frequency rTMS as a first-line treatment for gambling disorder – A case report

**DOI:** 10.1016/j.abrep.2025.100627

**Published:** 2025-08-05

**Authors:** Carmen Concerto, Fabrizio Bella, Cecilia Chiarenza, Ilenia White, Raffaele Micieli, Saverio Madonia, Stefania Lanzafame, Riccardo Spigarelli, Vera Pagano, Cristiano Cutuli, Fabio Brogna, Pasquale Caponnetto, Alessandro Rodolico, Maria Salvina Signorelli, Antonino Petralia

**Affiliations:** a Psychiatry Unit, Department of Clinical and Experimental Medicine, University of Catania, Catania 95124, Italy; b Oasi Research Institute - IRCCS, Via Conte Ruggero 73, Troina 94018, Italy; c Service for Addictions Unit, ASP Catania, Catania 95100, Italy; d Department of Educational Sciences, Section of Psychology University of Catania, Catania 95123, Italy; e Center of Excellence for the Acceleration of Harm Reduction (CoEHAR), University of Catania, Catania, Italy; f Technical University of Munich, TUM School of Medicine and Health, Department of Psychiatry and Psychotherapy, Klinikum rechts der Isar, Munich, Germany

**Keywords:** Gambling disorder, Transcranial magnetic stimulation, Neuromodulation, Addiction research, Case report

## Abstract

Gambling Disorder is a chronic and debilitating condition, often associated with impulsivity, mood disturbances, and significant social and financial consequences. Despite various pharmacological and psychotherapeutic approaches, standardized treatment is lacking. Repetitive transcranial magnetic stimulation (rTMS) has recently emerged as a potential neuromodulatory intervention for addictions. We report the case of a male patient in his sixties with severe Gambling Disorder who underwent TMS treatment with high-frequency rTMS targeting the left dorsolateral prefrontal cortex as a first-line therapy. The patient completed a six-week protocol. Clinical assessment was conducted at baseline, after six weeks, and after three months using multiple validated scales, including the South Oaks Gambling Screen, the Visual Analog Scale for craving, the Barratt Impulsiveness Scale, the Hamilton Anxiety and Depression Scales and the Canadian Problem Gambling Index. The patient’s South Oaks Gambling Screen score decreased from 20.5 to 2.0, and craving assessed via Visual Analog Scale reduced from 85 to 15 over three months. Reductions in anxiety, depression, and impulsivity were also observed, along with improvements in emotional stability and extroversion. Cognitive performance remained stable, and no adverse effects were reported throughout the treatment. This case highlights the potential of rTMS as a safe and effective intervention for Gambling Disorder, particularly in patients who are not willing to undergo a psychotherapy or pharmacological treatment. The progressive reduction in craving highlights the role of high rTMS in regulating compulsive behaviour and impulsive control.

## Highlights

• High-frequency rTMS was used as a first-line treatment for Gambling Disorder (GD).
• rTMS significantly reduced craving, impulsivity, anxiety, and depressive symptoms.
• Clinical improvements were maintained at 3-month follow-up without adverse effects.
• rTMS enhanced emotional stability and extroversion without cognitive side effects.
• This case supports rTMS as a safe, effective, non-pharmacological treatment for GD.

## 1 Introduction

In the Diagnostic and Statistical Manual of Mental Disorders (DSM)-5-TR, Gambling Disorder (GD) is classified under the “*Substance-Related and Addictive Disorders*” category (American Psychiatric Association, 2022). It is characterized by a persistent and problematic pattern of gambling behaviour that leads to significant distress or functional impairment. Key symptoms include persistent thoughts about gambling, a compulsion to bet increasing amounts of money, chasing losses, repeated unsuccessful attempts to stop, irritability when trying to quit, gambling to relieve distress, and lying to conceal the extent of the gambling problem (Potenza et al., 2019). Several risk factors have been identified for GD, including male gender, low educational attainment, lower socioeconomic status, and unemployment (Abbott, 2020). Moreover, GD is frequently associated with other psychiatric comorbidities, including Mood Disorders, Personality Disorders (particularly those in Cluster B), Substances Use Disorder and other behavioural disorders. Individuals affected by this condition are at a higher risk of suicide attempts, often linked to financial hardship or bankruptcy (Moreira et al., 2023). The aetiology and neurobiology of GD are complex and not yet fully understood. Both biological and environmental factors, like those found in Substance Use Disorder, contribute to the disorder (Linnet, 2020, Stefanovics et al., 2022). Key brain regions implicated include the frontostriatal and limbic networks—such as the orbitofrontal cortex, anterior cingulate cortex, insula, hippocampus, and amygdala. Studies showed that individuals with GD often exhibit reduced striatal activation during reward anticipation in response to gambling cues, alongside prefrontal network abnormalities affecting decision-making (Dufour et al., 2016). These neural dysfunctions underlie clinical features of GD, including increased reward sensitivity, executive dysfunction, impulsivity, stress dysregulation, and social–emotional issues (Del Mauro et al., 2023). Specifically, GD patients showed reduced striatal dopamine transporter availability, which is inversely related to the frequency of gambling and reward-based decision-making. Similarly, imaging studies in cocaine, heroin, and alcohol addiction revealed fewer dopamine receptors and reduced endogenous dopamine release, highlighting a “dopamine-impoverished” state in the addicted brain (Diana, 2011).

To date, various treatment approaches – including both psychosocial and pharmacological interventions – have been considered for GD, yet no standardized guidelines have been established (Stefanovics et al., 2022). While psychopharmacological agents, such as antidepressants and mood stabilizers, have yielded mixed results in improving GD outcomes, psychological therapies like cognitive-behavioural therapy and motivational interviewing have demonstrated efficacy in reducing gambling behaviours and related symptoms (Carlbring et al., 2010, Kraus et al., 2020, Ribeiro et al., 2021). However, the long-term durability of these therapeutic approaches remains uncertain, warranting further research (Di Nicola et al., 2020).

Recently, non-invasive brain stimulation techniques (NIBS) have attracted growing interest as potential treatments for GD, supported by promising findings in the treatment of Substance Use Disorder and other psychiatric conditions (Del Mauro et al., 2023, Ma et al., 2019, Torres-Castaño et al., 2021). Repetitive Transcranial Magnetic Stimulation (rTMS) is one of the most used NIBS (Zucchella et al., 2020). RTMS employs a magnetic field—generated by a coil placed on the scalp—to modulate neuronal excitability, potentially inducing both depolarization and hyperpolarization depending on stimulation parameters and cortical state. In fact, high-frequency protocols are generally associated with increased cortical excitability, while low-frequency protocols tend to have inhibitory effects (Edwards et al., 2019, Song et al., 2011). However, these effects are not universal, and accumulating evidence suggests that stimulation outcomes may vary depending on individual neurophysiological context and may involve mechanisms beyond simple LTP/LTD-like plasticity (Lefaucheur et al., 2020). High frequency rTMS primarily targeting the dorsolateral prefrontal cortex (DLPFC) has been shown to reduce craving by potentially increasing dopamine release in reward pathways and by improving prefrontal cortical function, thereby enhancing cognitive control (Gay et al., 2022). Indeed, the altered function of prefrontal areas, including the DLPFC, may account for impaired cognitive control, contributing to clinical aspects of GD, such as the progressive loss of control over gambling behaviours (Moccia et al., 2017). It has been shown that GD patients have reduced dopamine transporter availability in DLPFC, which is inversely related to the frequency of gambling and reward-based decision-making. These finding underscores dopamine dysregulation in DLPFC in individuals suffering from GD (Pettorruso et al., 2019). High-frequency stimulation targeting the left DLPFC was demonstrated to be effective in GD, with patients showing a substantial decrease in Gambling Symptom Assessment Score (GSAS) after rTMS treatment (Cardullo et al., 2019). Recent clinical trials indicate that rTMS is a safe and potentially effective treatment for GD, with clinically improvements observable after two weeks of treatment (Pettorruso et al., 2020). Previous studies reported clinical improvements in gambling symptoms after a single session of rTMS targeting the left DLPFC (Gay et al., 2017). These outcomes are likely attributable to striatal dopaminergic modulation exerted by high-frequency stimulation of the DLPFC, which is likely to be linked to the LTP and LTD-like phenomena (Cirillo et al., 2017). There are also several reports of the scarce effectiveness of rTMS in the treatment of GD, and further studies are needed (Concerto et al., 2023). Here, we present the case of a patient with GD who underwent high-frequency rTMS as a first-line treatment during an acute clinical episode, with escalating gambling behaviour, intense craving and significant functional impairment. Through a detailed analysis of the clinical outcomes of rTMS treatment, we aimed to contribute to the understanding of the potential of this therapeutic approach. This case report was prepared in accordance with the ethical standards of the institutional and national research committee and with the 1964 Helsinki declaration and its later amendments. The intervention was part of standard clinical care. This case report is presented to highlight the real-world application of rTMS as a first-line, stand-alone intervention for GD, in the absence of pharmacological or psychotherapeutic treatments. While several clinical studies have previously evaluated rTMS in GD (Gay et al., 2017, Pettorruso et al., 2020, Rosenberg et al., 2013, Sauvaget et al., 2018, Zack et al., 2016), these were typically conducted in controlled research settings, often in combination with other treatments. In contrast, the present case offers a detailed psychometric and functional follow-up in a patient who refused medication and was treated during an acute clinical phase. The rationale for reporting this single case lies in its clinical novelty, ethical implications, and potential translational relevance, particularly for patients who are not eligible or willing to undergo standard therapies.

### 1.1 Case presentation

A male patient in his sixties, working in a creative field and residing in southern Italy presented to our clinic seeking treatment for a ten-year history of pathological gambling and impulsive behaviours. The patient reported a progressive escalation of maladaptive behavioural patterns that significantly affected his personal and financial well-being. According to his account, symptoms began roughly a decade prior with increasing gambling behaviour. Impulsive shopping and excessive spending emerged gradually during the second year, suggesting escalating reward-seeking behaviour rather than manic episodes. No other affective switches or elevated mood episodes were reported. To corroborate the severity of his impulsive behaviour, the patient produced elaborate lies to justify these purchases to his family. The gambling and impulsive behaviours led to severe financial consequences, including a depletion of his bank accounts, pawning of family gold, and borrowing money from friends and relatives. The patient denied any history of substance use, alcohol consumption, psychosis, mania nor prior psychiatric treatments. The patient had previously completed a 6-month course of cognitive-behavioural psychotherapy with a private specialist approximately five years prior to our evaluation. However, this intervention did not result in any significant clinical improvement. The patient was married and lived with his wife. He maintained limited but stable social contacts, primarily with a few close friends. There was no history of psychiatric illness, substance use, or behavioral disorders in first-degree relatives. The patient's family environment was described as emotionally distant, but without reports of trauma or abuse. This context likely contributed to the development of an avoidant attachment style, as also reflected in the psychometric findings.

In November 2024, the patient underwent an outpatient clinical evaluation at the Psychiatric Unit of the “G. Rodolico” Policlinico Hospital of Catania, Italy. The patient showed intact cognitive functioning, mild anxiety, and a slightly depressed mood. Following a clinical assessment, the patient met the DSM-5-TR criteria for GD. Given the psychopathological acute phase characterized by increased craving levels, uncontrolled gambling behaviour and impaired social functioning, a pharmacological treatment was proposed, which the patient declined. This refusal appeared to be linked to the fear of potential side effects associated with medications. The patient was not taking any other medications, and no significant psychiatric or medical comorbidities were identified. For this reason, we proposed a non-invasive somatic treatment with TMS, which the patient readily accepted, reporting he felt more comfortable with this approach rather than a pharmacological one.

The patient underwent a comprehensive psychological and neuropsychological assessment (Table 1) at three-time points: before initiating treatment (T0), at 6 weeks (T1) and 3 months after treatment (T3). At baseline, South Oaks Gambling Screen (SOGS) revealed a severe pattern of disordered gambling (20 ± 1.5 SD, maximum score: 20 points): the patient consistently reported behaviours such as gambling with more money than he could afford, repeatedly attempting to recover losses (“chasing”), borrowing money or selling possessions to finance gambling, and experiencing significant health, stress, and anxiety issues related to gambling. Additionally, the patient acknowledged feeling guilty regarding his gambling behaviour and reported frequent criticism from family members due to gambling-related financial problems. These findings indicated a loss of control, financial harm, and psychological distress, all of which are hallmark features of GD. The Canadian Problem Gambling Index (CPGI) yielded a score of 22 ± 1.0 SD (maximum score: 27 points), which corroborated a severe gambling problem in accordance with SOGS results. Obsessive-compulsive symptoms were minimal, as assessed by the Yale-Brown Obsessive-Compulsive Scale (Y-BOCS) (1 ± 0.5 SD, maximum score: 40 points). Personality assessment with the Big Five Inventory (BFI) highlighted notable traits: high agreeableness (4.43 ± 0.49 SD), indicating an empathetic and cooperative nature; high conscientiousness (4.14 ± 0.69 SD), reflecting reliability and organisation; and exceptional openness to experience (4.71 ± 0.49 SD), signifying creativity and curiosity. The patient also demonstrated moderate extroversion (3.3 ± 0.5 SD) and low neuroticism (2.8 ± 0.4 SD). Impulsivity, assessed through the Barratt Impulsiveness Scale (BIS-11), scored 64 ± 3.0 SD (maximum score: 120 points), indicating low-moderate impulsivity. Attachment style evaluation using the Relationship Questionnaire revealed a predominant avoidant attachment (Type C: 6 points), associated with anxiety about relationships and low self-worth.

**Table 1:** Psychodiagnostic assessment at baseline, at the end of treatment and at three months follow-up. Values reflect mean ± standard deviation calculated from item-level responses; standard deviations represent intra-scale variability within the single subject. The SF-12 is reported based on patient self-assessment rather than numerical scoring.

Questionnaires	Baseline (Mean ± SD)	End of treatment (Mean ± SD)	Follow-up (Mean ± SD)
SOGS	20 ± 1.5	7.0 ± 2.0	2.0 ± 1.0
VAS	85 ± 5	45 ± 7	15 ± 5
Y-BOCS	1.0 ± 0.5	0.0 ± 0	0.0 ± 0
BIS-11	64 ± 3	56 ± 4	48 ± 3
HAM-A	11 ± 2	4 ± 1	2 ± 1
HAM-D	16 ± 3	6 ± 2	1 ± 1
BFI − Neuroticism	2.8 ± 0.4	2.0 ± 0.3	1.5 ± 0.2
BFI − Extroversion	3.3 ± 0.5	3.5 ± 0.4	3.9 ± 0.3
ISI	2.0 ± 0.8	1.0 ± 0.5	0.5 ± 0.2
SF-12	*Moderate (subjective)*	*Improved (subjective)*	*Very good (subjective)*
CPGI	22.0 ± 1.0	9.0 ± 1.5	3.0 ± 1.0
MoCA	26.0 ± 1.0	27.0 ± 0.5	28.0 ± 0.5

Neuropsychological testing indicated preserved cognitive functioning, with a Montreal Cognitive Assessment (MoCA) score of 26 ± 1.0 SD (maximum score: 30 points), within the normal range. Mild signs of depression were observed across multiple scales, including the PHQ-9 (9 ± 0.78 SD, maximum score: 27 points), Beck Depression Inventory-II (3 ± 0.73 SD, maximum score: 63 points), and the Hamilton Depression Rating Scale (HAM-D) (16 ± 3.0 SD, maximum score: 52 points). A mild executive functioning deficit, clinically not significant, was noted on the Frontal Assessment Battery (FAB) (17 ± 0.41 SD, maximum score: 18 points). Anxiety levels, as measured by the Hamilton Anxiety Scale (HAM-A) were mild (11 ± 2.0 SD, maximum score: 56 points), and no clinically significant sleep disturbances were observed, as indicated by the Insomnia Severity Index (2 ± 0.8 SD, maximum score: 28 points). Also, the patient reported a very high level of craving for gambling, as assessed through the Visual Analog Scale (VAS) which scored 85 ± 5.0 SD (maximum score: 100 points). This finding indicated a nearly uncontrollable urge to gamble, consistent with the severe compulsive behaviour and impulsivity observed in the psychometric evaluations.

## 2 Methods

The patient underwent a high frequency rTMS protocol targeting the left DLPFC. originally approved for depression treatment (O’Reardon et al., 2007). The same protocol has been previously used in GD research (Gay et al., 2017, Sauvaget et al., 2018, Zucchella et al., 2020). Specifically, the protocol was performed over 6 weeks, for a total of 30 sessions of high-frequency rTMS targeting the left DLPFC (10 Hz; 120 % of RMT, 50 trains of 40 pulses with 26 s inter-train interval; 1/daily for 5 consecutive days). The total duration of the session was 37 min, and the total number of pulses was 3000 for each session. A MagStim Rapid 2 device (Magstim Co., Whitland, Wales, UK) equipped with a Magstim D70 mm Air Film Coil (Magstim Co., Whitland, Wales, UK) was used for the TMS treatment that was delivered by trained medical doctors. The resting motor threshold (RMT) was measured by stimulating the First Dorsal Interosseous (FDI) motor hotspot area and by visually observing twitch responses in the contralateral hand. The stimulation intensity that evoked visible FDI movement in 5 out of 10 pulses was considered as the RMT (Badran et al., 2019, Rossini et al., 2015). The DLPFC site was identified using the F3/F4 method for locating the F3 position from the EEG 10/20 international system. The patient did not complain of any adverse effects attributable to the stimulation during the entire duration of the treatment, which the patient reported to be well tolerated. This was assessed through clinical interviews conducted before each treatment session. All procedures followed ethical standards. The patient provided written informed consent for the publication of all clinical and psychometric data reported in this article. All personally identifying information was removed or anonymized to ensure full confidentiality.

## 3 Results

Six weeks after rTMS treatment, the patient was reassessed using the same scales, revealing clinically relevant improvements (Table 1). The comparison between the baseline and six weeks follow-up SOGS scores (7.0 ± 2.0 SD) showed that the patient’s tendency to gamble with more money than he could afford was greatly reduced. Also, the need to gamble larger amounts to achieve the same level of excitement diminished, and even chasing losses, which was previously frequent, became occasional or absent. The patient no longer relied on borrowing money or selling personal items to finance gambling, with feelings of guilt related to gambling behaviour substantially decreased. The patient also reported fewer conflicts and discussions with family members regarding money or gambling and, overall, the negative impact of gambling on health, stress levels, and family finances was significantly reduced. Importantly, the patient-maintained awareness of having had a gambling problem in the past, but no longer demonstrated behaviours indicative of active GD. After six weeks, the patient’s craving substantially decreased, as shown by the VAS score (45 ± 7.0 SD). This moderate level of craving reflected the partial therapeutic response and aligned with the reduction in depressive symptoms, impulsivity, and gambling behaviour measured by the other scales. Although the urge to gamble was still present, the patient demonstrated an increased ability to resist impulses and maintain control. Furthermore, the patient obsessive–compulsive symptoms were resolved, as evidenced also by a decrease in the Y-BOCS score (0 ± 0 SD). Impulsivity levels, measured by the BIS-11, was reduced moving into a lower impulsivity range (56 ± 4.0 SD). Anxiety levels, assessed by the HAM-A, dropped significantly (4 ± 1.0 SD), indicating minimal anxiety. Depressive symptoms also improved significantly, with the HAM-D score reducing (6 ± 2.0 SD). The Big Five Inventory demonstrated subtle changes in personality traits. Emotional stability improved, indicating enhanced stress management and emotional resilience. Extroversion showed a mild increase, while agreeableness and openness to experience remained high. Cognitive functioning remained stable, and sleep patterns showed minimal improvement.

To assess the medium-term benefits of the treatment provided, the patient underwent a follow-up assessment three months after baseline. SOGS showed near-complete remission of gambling behaviours, with all key indicators returning to negative responses (2 ± 1.0 SD). The patient no longer reported hiding activities, asking for loans, or experiencing gambling-induced distress. The VAS score further decreased, indicating minimal or absent craving (15 ± 5.0 SD). This outcome aligned with improvements across all the other assessments. The patient no longer reported intrusive gambling thoughts or the compulsion to gamble, and the urge was perceived as occasional and easily manageable. He also referred near-normal sleep, with occasional minor difficulties and no anxiety associated with rest as shown by ISI score (0.5 ± 0.2 SD). Y-BOCS revealed that compulsive thoughts were rare, with good impulse control and minimal associated distress (0 ± 0 SD). Furthermore, the assessment documented no interference in daily activities, better emotional balance, and restored concentration abilities. BFI results showed increased self-confidence, greater extroversion, openness to experience, and reduced neuroticism, indicating improved emotional regulation and social functioning. At this 3-month follow-up, there was also a marked reduction in impulsivity levels, enhanced planning ability, and better impulse management as demonstrated by BIS-11 (48 ± 3.0 SD). HAM-D scores reflected near-complete remission (1 ± 1.0 SD), with improved mood stability, normalized sleep, and absence of somatic and cognitive anxiety symptoms (Table 1).

## 4 Discussion

In this case report we explored the effect of a high-frequency rTMS protocol for the treatment of GD symptoms. This therapeutic approach was chosen in response to the clinical necessity of treating a patient with severe GD during an acute phase of the disease who declined pharmacological intervention. The 6-week course of rTMS yielded a substantial reduction in craving alongside marked improvements in other clinical dimensions of GD. Importantly, the intervention was well tolerated, with no treatment-related adverse effects observed.

**Fig. 1:**
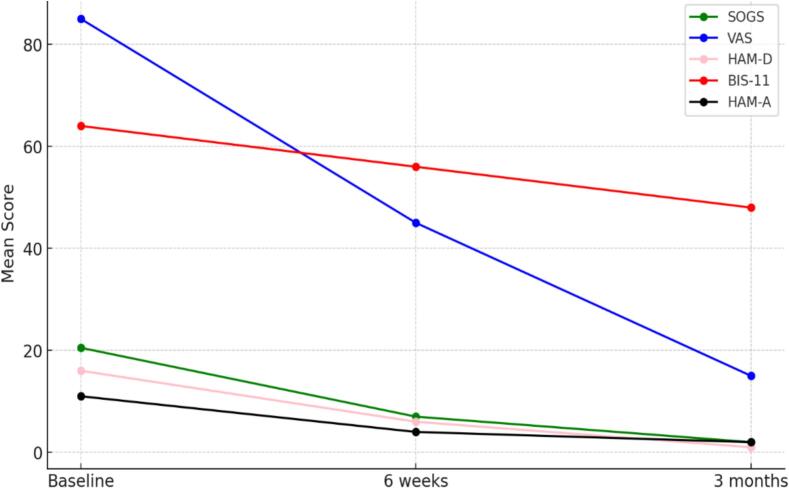
Improvements in craving, impulsivity, anxiety and depressive symptoms over time (from Baseline to 3 Months).

This case report provides a unique insight into the use of TMS as a first-line treatment for GD, demonstrating its potential efficacy in mitigating craving, impulsivity, anxiety, and depressive symptoms associated with the condition (Fig. 1). To our knowledge, this is one of the few documented cases where TMS has been applied as a primary intervention for GD, highlighting its therapeutic potential in a field that remains underexplored.

The choice of rTMS as the sole intervention was dictated by the patient’s refusal to take pharmacological treatments, a common barrier in GD management. This underscores the importance of offering alternative strategies, non-pharmacological therapies to address the diverse needs of GD patients. Of particular importance is the absence of adverse effects or tolerability issues, reaffirming the safety profile of rTMS. The patient reported improvements in daily functioning and perceived quality of life, underscoring the clinical relevance of this intervention beyond standard psychometric scores.

The patient exhibited substantial clinical improvements across multiple domains. Impulsivity, a core feature of GD, was substantially reduced, as reflected in the Barratt Impulsiveness Scale score. This outcome aligns with prior findings indicating that TMS over the DLPFC can modulate neural circuits associated with impulsivity and decision-making, which are often dysregulated in behavioural addictions (Koob & Volkow, 2016). The reduction in anxiety and depressive symptoms further supports the role of DLPFC stimulation in enhancing emotional regulation, as previously demonstrated in major depressive disorder studies (Concerto et al., 2015, Sonmez et al., 2019).

Beyond symptom reduction, this case also highlights subtle yet noteworthy effects in personality dimensions, particularly improved emotional stability and extroversion. These findings raise intriguing questions about whether rTMS can induce neuroplastic changes that extend beyond symptom control to influence broader psychological traits and coping mechanisms. Furthermore, the patient’s avoidance attachment style, identified during baseline assessments, raises questions about the interplay between personality traits and GD. Avoidant attachment is often linked to difficulties in emotional expression and a predisposition to maladaptive coping strategies, such as gambling. While TMS primarily targets neural mechanisms, the observed improvements in emotional stability and impulsivity suggest that TMS may also have indirect benefits on attachment-related behaviours.

Although several prior studies have explored the efficacy of rTMS in Gambling Disorder (Cardullo et al., 2019, Pettorruso et al., 2019, Salerno et al., 2022), the present case report offers clinically meaningful insights by documenting the use of rTMS as a first-line monotherapy in a naturalistic setting. Unlike controlled trials, which often involve pharmacological or psychotherapeutic co-interventions, this case involved a treatment-naïve patient who declined standard pharmacotherapy. The patient was treated during an acute clinical exacerbation, and the intervention resulted in sustained improvement over a three-month period, as assessed by comprehensive psychometric evaluations. While the absence of mechanistic data (e.g., neuroimaging or neurophysiological measures) is acknowledged, the strength of this case lies in its practical relevance, ethical dimension, and potential to inform clinicians dealing with similarly complex presentations. This report may also help generate hypotheses and support the rationale for future controlled studies focusing on non-pharmacological interventions in GD.

Despite these promising findings, this case report has limitations. First, the outcomes are based on a single patient, limiting the generalizability of the results. Second, the absence of a placebo or control group precludes definitive conclusions about the causal effects of TMS. Lastly, the improvements in personality traits, while noteworthy, warrant further investigation to determine whether these changes are directly attributable to TMS or they reflect a broader behavioural adjustment. Additionally, while the improvements are promising, the lack of long-term follow-up data leaves unanswered questions about relapse risk and the durability of therapeutic benefits. Future research should focus on randomized controlled trials and incorporate neurophysiological measures such as functional MRI or EEG to better understand the neural correlates of clinical improvement. Additionally, investigating combined interventions (rTMS paired with cognitive-behavioural therapy) may yield synergistic effects and further enhance treatment efficacy.

## 5 Conclusions

To the best of our knowledge, this is the first case report that investigated the use of a 6-week high-frequency rTMS protocol as a stand-alone intervention. Unlike single-session or short-term protocols explored in previous studies, our extended treatment course allowed us to evaluate the cumulative and sustained clinical effects of rTMS over time. The patient's marked improvements across multiple domains underline the potential of rTMS as a viable, non-pharmacological alternative for patients who refuse or cannot tolerate conventional treatments.

Future studies should aim to explore the long-term efficacy of TMS in GD, including its impact on relapse rates and quality of life. Additionally, integrating TMS with cognitive-behavioural therapies could enhance its therapeutic potential by addressing both the neurobiological and psychological dimensions of GD.

In conclusion, this case underscores the potential of TMS as a safe and effective treatment for GD, particularly in patients who are unable to tolerate pharmacological interventions.

The observed clinical improvements provide an encouraging rationale for further investigation. As suggested by psychological assessment, the patient’s progression over three months highlights substantial clinical recovery from GD, accompanied by improvements in sleep, psychological well-being, and functional capacity.

By targeting the neural circuits implicated in impulsivity and emotional dysregulation, TMS offers a novel approach to address the complex neurobehavioral underpinnings of GD. Further research is warranted to validate these findings and expand the therapeutic landscape for this debilitating condition.

## CRediT authorship contribution statement

**Carmen Concerto:** Writing – review & editing, Supervision, Project administration, Methodology, Investigation, Data curation, Conceptualization. **Fabrizio Bella:** Writing – review & editing, Writing – original draft, Investigation. **Cecilia Chiarenza:** Investigation. **Ilenia White:** Investigation. **Raffaele Micieli:** Investigation. **Saverio Madonia:** Investigation. **Stefania Lanzafame:** Investigation. **Riccardo Spigarelli:** Investigation. **Vera Pagano:** Investigation. **Cristiano Cutuli:** Investigation. **Fabio Brogna:** Investigation. **Pasquale Caponnetto:** Investigation. **Alessandro Rodolico:** Writing – review & editing. **Maria Salvina Signorelli:** Supervision. **Antonino Petralia:** Supervision.

## Funding

The research received no external funding.

## Declaration of competing interest

The authors declare that they have no known competing financial interests or personal relationships that could have appeared to influence the work reported in this paper.

## Data Availability

Data will be made available on request.
